# Solitary neurofibroma of the right lateral wall of the oropharynx

**DOI:** 10.4314/gmj.v55i3.11

**Published:** 2021-09

**Authors:** Winga Foma, Pani Awesso, Essobozou P Pegbessou, Bathokedeou Amana

**Affiliations:** 1 Department of ENT, Sylvanus Olympio Teaching Hospital of Lomé, Togo; 2 Department of ENT, Prefectural Hospital of Tsevié, Togo; 3 Department of ENT, Campus Teaching Hospital of Lomé, Togo

**Keywords:** neurofibroma, solitary neurofibroma, oropharynx, dysphagia

## Abstract

Solitary neurofibroma of the oropharynx is extremely rare. Imaging explorations may be necessary, but the diagnostic certainty is pathological. We report a case of benign tumour of the oropharynx in a 25-year-old woman who was seen for a consultation with dysphagia, a change in voice and dyspnea in the supine position. The excision was performed under general anaesthesia with orotracheal intubation via the oropharyngeal route. Pathological examination of the surgical specimen revealed neurofibroma. Although rare, solitary neurofibroma of the oropharynx should be considered in any benign tumour in the area.

## Introduction

Neurofibroma is a benign tumour of the peripheral nerves, most often isolated but sometimes multiple, especially in neurofibromatosis. Symptoms vary depending on the location. Oropharyngeal localization is extremely rare and can manifest as simple pharyngeal discomfort, dysphagia, a covered voice or even difficulty breathing. The objective of this presentation was to report a rare case of symptomatic solitary neurofibroma of the oropharynx in a young woman and discuss its management in our context.

## Case Report

**Figure 1:**
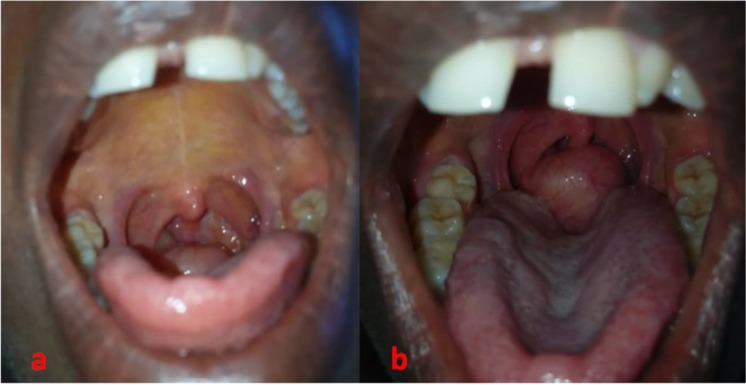
Oropharyngeal mass covered with a normallooking mucosa (a) arising from the right lateral oropharynx and ascending to the protraction of the tongue (b).

**Figure 2:**
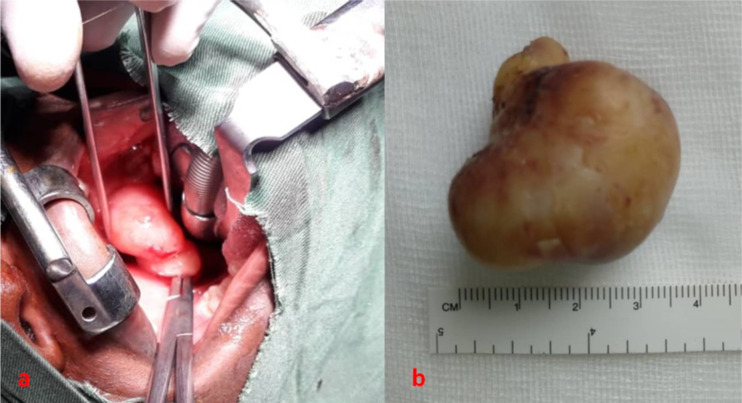
Intraoperative view with lesion pedicle clamped with hemostasis forceps (a), surgical specimen (b).

This was a 25-year-old housewife who was seen for a consultation with dysphagia to solids developing for eight months of gradual onset. Four months after the onset of dysphagia, there had been a change in voice and dyspnea in the supine position. The ENT examination showed at the mouth opening a tumour covered with a normal-appearing mucosa from the right lateral oropharynx, which ascended to the protraction of the tongue (Figures 1a and 1b), firm on palpation. Indirect nasofibroscope laryngoscopy showed a pedicled oval tumour hanging from the right lateral wall of the oropharynx behind the posterior pillar. The pre-surgical laboratory workup was unremarkable. The excision was performed under general anaesthesia with orotracheal intubation via the oropharyngeal route (Figure 2a). After an incision of the mucosa at the base of the pedicle, it was dissected using a stripper and then resected. The closure was made with three stitches.

The surgical specimen measured 4cm x3cm x 3cm (figure 2b).

**Figure 3:**
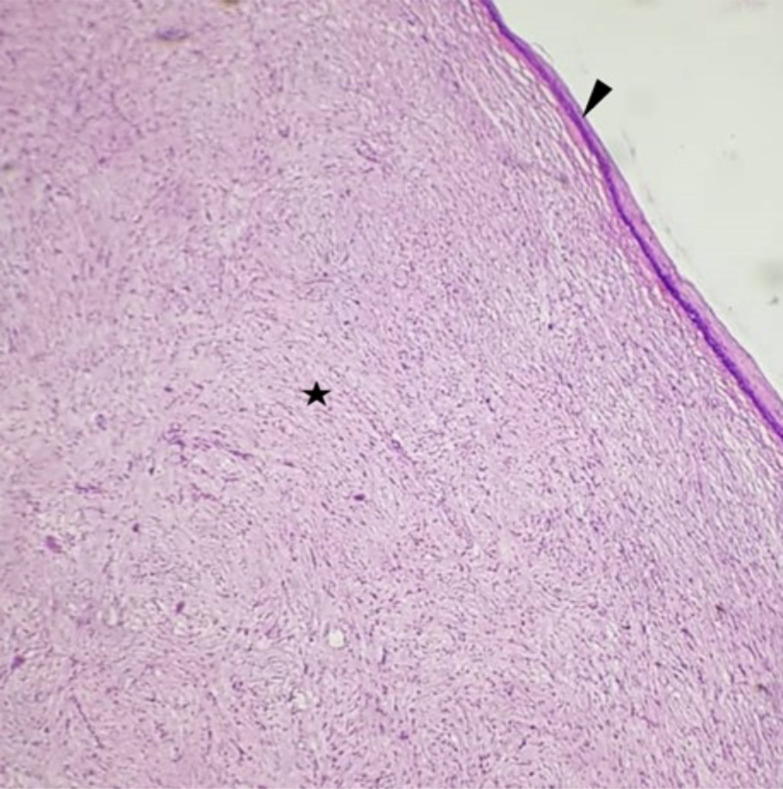
Histological appearance of the neurofibroma: Note under a normal, metaplastic squamous epithelium (arrowhead), a spindle cell tumour with wavy nuclei (star) (HE; x10).

Pathological examination of the surgical specimen revealed neurofibroma (Figure 3). Faced with this diagnosis, the search for signs favouring neurofibromatosis, including café-au-lait spots on the body, was unremarkable. The patient reported no sensory or motor disorder one year after the operation.

## Discussion

Neurofibroma is a benign tumour that can arise from cells of the Schwann sheath, such as schwannomas, but also endoneural, perineural or fibroblast cells.^1^,^2^ The neurofibroma, whether solitary, diffuse or plexiform, rather occurs in young adults, between 20 and 30 years old, without sexual predominance.^3^,^4^ Clinical symptomatology involves the oropharynx, including dysphagia, dyspnea and sleep apnea syndrome.^4^ On clinical examination, its surface is smooth, sometimes polypoid. Sensory nervous disorders and pain rarely accompany the lesion.

Computed tomography determines the site and the limits of the tumour and reveals a tissue mass with heterogeneous contrast enhancement characteristic of neurofibroma^5^ MRI is the reference radiological examination in front of a neurofibroma, it shows a hypointense in T1 and a hypersignal in T2, and when it is voluminous, it may contain a central hyposignal producing a characteristic cockade appearance.^3^,^5^ In our case, the benign and pedunculated nature of the lesion favoured endoscopic surgical exploration without going through other costly paraclinical explorations for the patient. The definitive diagnosis is based on the pathological examination of the surgical specimen or a biopsy.

The management of neurofibroma is essentially surgical and helps prevent possible degeneration. Due to the close connection of the neurofibromatous lesion with normal nervous tissue, its complete surgical resection exposes neurological deficits in the case of a major nerve.^4^ The simple surgical consequences without an oropharyngeal neurological deficit in the present observation show the minor nature of the nervous element involved.

## Conclusion

Although rare, solitary neurofibroma of the oropharynx should be considered for any benign tumour in the area. Imaging explorations may be necessary, but the diagnostic certainty is pathological. Surgical management prevents the occurrence of malignant degeneration.
